# Embryonic and Neonatal Mouse Cochleae Are Susceptible to Zika Virus Infection

**DOI:** 10.3390/v13091823

**Published:** 2021-09-14

**Authors:** Vidhya Munnamalai, Nabilah H. Sammudin, Caryl A. Young, Ankita Thawani, Richard J. Kuhn, Donna M. Fekete

**Affiliations:** 1 The Jackson Laboratory, Bar Harbor, ME 04609, USA; Vidhya.Munnamalai@jax.org (V.M.); Caryl.Young@jax.org (C.A.Y.); 2 Graduate School of Biomedical Sciences and Engineering, University of Main, Orono, ME 04469, USA; 3 Department of Biological Sciences, Purdue University, West Lafayette, IN 47907, USA; nchesamm@uchicago.edu (N.H.S.); ankita.thawani@bcm.edu (A.T.); kuhnr@purdue.edu (R.J.K.); 4 Purdue Institute of Inflammation, Immunology and Infectious Disease, Purdue University, West Lafayette, IN 47907, USA; 5 Purdue Institute for Integrative Neuroscience, Purdue University, West Lafayette, IN 47907, USA

**Keywords:** ZIKV, cochlea, hair cell, hearing

## Abstract

Congenital Zika Syndrome (CZS) is caused by vertical transmission of Zika virus (ZIKV) to the gestating human fetus. A subset of CZS microcephalic infants present with reduced otoacoustic emissions; this test screens for hearing loss originating in the cochlea. This observation leads to the question of whether mammalian cochlear tissues are susceptible to infection by ZIKV during development. To address this question using a mouse model, the sensory cochlea was explanted at proliferative, newly post-mitotic or maturing stages. ZIKV was added for the first 24 h and organs cultured for up to 6 days to allow for cell differentiation. Results showed that ZIKV can robustly infect proliferating sensory progenitors, as well as post-mitotic hair cells and supporting cells. Virus neutralization using ZIKV-117 antibody blocked cochlear infection. AXL is a cell surface molecule known to enhance the attachment of flavivirus to host cells. While *Axl* mRNA is widely expressed in embryonic cochlear tissues susceptible to ZIKV infection, it is selectively downregulated in the post-mitotic sensory organ by E15.5, even though these cells remain infectible. These findings may offer insights into which target cells could potentially contribute to hearing loss resulting from fetal exposure to ZIKV in humans.

## 1. Introduction

Zika virus (ZIKV) is a flavivirus that received world-wide attention in 2015 when a Brazilian outbreak revealed that ZIKV infection of pregnant women was linked to severe birth defects in their newborns [1]. In the wake of the South American epidemic, Congenital ZIKV Syndrome was defined by a set of pathologies that included severe microcephaly, thin cerebral cortices with brain calcifications, eye defects, limb contractures and hypertonia as major diagnostic criteria [2]. Hearing loss was linked to in utero ZIKV exposure in 12% of 114 infants evaluated during the second year of life [3]. A review of 27 studies that looked for a positive correlation between fetal ZIKV exposure and congenital hearing loss shows a wide range in this co-morbidity [4]. Non-invasive screening for hearing loss in infants utilizes two methods that interrogate different parts of the auditory system (reviewed by [5,6,7]). The auditory brainstem response (ABR) records neuronal responses to sound stimuli and provides threshold readouts for neurons from the auditory nerve through to the midbrain. Otoacoustic emission (OAE) can identify defects in the peripheral auditory system, particularly in the outer hair cells of the organ of Corti (the sensory organ for hearing in a mammalian inner ear). Outer hair cell motility can transmit energy from the cochlea through the middle ear to vibrate the tympanic membrane. This leads to sounds being emitted from the ear that are detected by sensitive microphones placed in the ear canal. Thus, an ear presenting with weak or absent OAEs is suspected to have defective or missing outer hair cells as an underlying cause [8]. In the context of ZIKV infection, this raises the question of whether the virus can cause hair cell pathologies as a sequela of direct infection of these primary sensory receptors.

The susceptibility of the inner ear tissues to ZIKV was recently reported for two vertebrate animal models: chicken embryos [9] and adult mice with compromised immune systems [10]. In the chicken embryo, our group found that the inner ear could be infected by direct delivery of virus into the fluid compartment of the inner ear. The outcome from this route of infection varied for different cell types (such as neurons, hair cells and semicircular canals). For instance, the auditory ganglion was most susceptible to infection during the first week in ovo, when inner ear progenitors are mitotically active, and responded with an increase of cell death and major shrinkage. In contrast, the sensory organ of hearing was more susceptible to ZIKV during the second week in ovo, during hair cell maturation, but this infection did not generate overt pathology over several days. This suggests that the different inner ear cell types may have specific critical periods of susceptibility and sensitivity to ZIKV, although until now this has not been tested in an embryonic mammalian inner ear. In the 5−6-week-old immunocompromised mouse, systemic ZIKV delivery resulted in infection of the inner ears as well as subsequent histological pathologies at 9 days post-infection [10]. Cellular damage (swelling, intracellular vacuoles, translocation of the nucleus and/or apical surface blebbing) was observed in the sensory epithelia (both auditory and vestibular) or in the spiral ganglion, whose axons bundle to form the auditory nerve. Other regions of the cochlea, such as the stria vascularis, displayed epithelial detachment. Our current study aimed to determine if the mammalian cochlea is equally vulnerable to ZIKV during its development. In view of reports that mitotically active neural progenitors are more susceptible to ZIKV infection than post-mitotic neurons [11], this study examined stages of cochlear development that spanned the time from mitotic to postmitotic stages. These experiments can guide neonatal diagnostics by establishing a timeline of susceptibility of the cochlear duct.

Recent studies have identified antibodies directed against ZIKV that can neutralize its ability to infect mammalian host cells [12,13], with the goal toward understanding binding of the virus, as well as seeking potential therapeutic agents to combat ZIKV spread [14,15]. In this study, we also explore the possibility of neutralizing active ZIKV infection of the cochlea with a known ZIKV-specific antibody, ZIKV-117.

An active area of investigation is the search for cellular receptors and mechanisms used by ZIKV to attach to host cells, gain entry into the cell and initiate a productive infection. AXL is a member of the TYRO3/AXL/MER (TAM) family of cell-surface proteins that can promote ZIKV entry/attachment in some human and mouse cells (reviewed by [16]). While AXL is not strictly required for ZIKV to initiate a productive infection in mice, its tissue distribution can sometimes be correlated with variable sensitivity to ZIKV. Here, we also look at *Axl* expression as a function of developmental stage in the embryonic and neonatal mouse cochlea, to seek a correlation between *Axl* localization and tissue susceptibility to ZIKV.

## 2. Materials and Methods

### 2.1. Mice

Swiss Webster mice (Envigo, Inc., Indianapolis, IN, USA) were housed and bred in accordance with the Institutional Animal Care and Use Committee (IACUC) at Purdue University and The Jackson Laboratory (ACUC). Veterinary care and oversight of the animal facilities and husbandry practices were provided at The Jackson Laboratory and the Laboratory Animal Program (LAP) at Purdue University.

### 2.2. Collection of Embryos and Neonatal Pups

Mice were timed mated and the date at which a vaginal plug was observed was noted as embryonic day 0.5 (E0.5). To harvest embryos of defined ages (E12.5–E18.5), pregnant mice were euthanized via CO_2_ inhalation, followed by secondary cervical dislocation. Embryos were extracted from the uterus and placed in cold Hank’s Balanced Salt Solution (HBSS). The developmental age of the litter was determined via Theiler staging: http://www.emouseatlas.org/emap/ema/theiler_stages/downloads/theiler2.pdf (accessed 1 June 2016). Embryos were then decapitated. Postnatal day 2 (P2) pups were anesthetized via hypothermia on ice, followed by rapid decapitation. The heads from embryos and pups were either dissected to isolate the cochleae for organ culture or they were placed into 4% paraformaldehyde (PFA) in phosphate-buffered saline (PBS) for histological processing.

### 2.3. Cochlear Cultures

These experiments were started with ZIKV virus stocks of the Asian lineage (H/PF/2013) titered at 8.7–9.6 × 10^7^ plaque-forming units per milliliter (PFU/mL). These were aliquoted into smaller volumes to reduce freeze-thaw cycles for the subsequent experiments. Aliquots were stored at −80 °C and thawed on ice immediately before adding 5 microliters (~5 × 10^5^ PFU) to each filter. Approximately 2 years later, the titers had dropped to 1.5–1.7 × 10^7^ PFU/mL. Thereafter, to compensate for these lower titers, we increased the inoculum to 8 microliters (~10^5^ PFU) per filter for experiments conducted over the final year of the study. Most of these latter experiments were included in Experimental Group B described below.

Cochleae from staged embryos were dissected in HBSS and cultured with their apical surfaces up by placing on Collagen/Matrigel-coated Millicell culture inserts (Invitrogen, Inc.) at 37 °C in a 4% CO_2_ incubator as described [17]. For cultures initiated on E12.5, the cochlear duct was usually not opened, as we had determined that virus could adequately diffuse into these smaller ducts. For cultures started on E15.5 or P2, the cochlear duct was deliberately opened to expose the apical surface of the sensory epithelium. Two to four cochleae (with or without the saccular macula attached) were placed on each filter.

After culturing for 4–6 h to allow the explants to adhere, ZIKV (1–5 × 10^5^ PFU) was dripped onto the top of each filter and they were returned to the incubator. After 24−48 h, the medium was replaced and cochleae were incubated for several additional days to allow sensory hair cells and supporting cells to begin to differentiate, recognizing that ZIKV might continue to spread during this time window. Approximately equal numbers of cochleae were untreated to serve as negative controls. Cochleae were cultured for either 3 or 6 days in vitro (DIV).

Experimental Group A was used for determination of cell types that were infected with ZIKV and cultured for 6 DIV. This included experiments initiated at E12.5 (*n* = 3 experiments), E15.5 (*n* = 4) and P2 (*n* = 3) with ZIKV added for 24 h. For three additional experiments, virus was left in the cultures for 48 h before washout (Supplementary Table S1). In total, 93 organs, including controls, were cultured and evaluated in Experimental Group A.

Experimental Group B was used to evaluate cell death in control and ZIKV-exposure organs. This group included 6 experiments, with a set of E15.5 plus 3 DIV (*n* = 4) used for quantitative analysis of active Caspase-3. Experimental Group B comprised 41 organs.

Experimental Group C was used for antibody blocking experiments using ZIKA-117 that neutralizes Zika virus replication [12]. To make sure the neutralizing antibody had full access to the sensory organs at the time of explantation on E12.5, the roofs of the ducts were removed prior to being placed on inserts. Selected explants were pre-incubated with 5 μL of ZIKV-117 antibody (8.2 μg/μL). At 6 h, ZIKV was added directly on top of the explants and both the antibody and virus were washed out after 24 h. A total of 19 cochleae were cultured for 6 DIV and evaluated for Experimental Group C.

For all 3 experimental groups, the cochleae were fixed with 4% PFA in PBS and processed for immunofluorescence as previously described [18]. The data presented here are based on 21 separate culture experiments (i.e., set up on different days) totaling 153 explants (Supplementary Table S1).

### 2.4. Histology

Primary antibodies were used to detect double-stranded RNA, dsRNA (1:500; mouse IgG_2a_, J2 monoclonal, SCICONS #10010200), active Caspase-3 (1:400; rabbit IgG; BD Biosciences #559565), Sox2 (1:100–1:250 goat polyclonal, R&D Systems #AF2018) and myosinVI (1:200; rabbit polyclonal; Proteus Biosciences #25-6791). Alexa Fluor^®^ secondary antibodies (1:500, donkey or goat polyclonal; Invitrogen) of 488 (green), 568 (red) or 647 (far red) varieties were chosen as appropriate to allow for triple-labeling.

For in situ hybridization, heads were fixed overnight in RNase-free 4% PFA in PBS, moved through graded sucrose solutions (10%, 20%, 30% in PBS) for several hours to overnight, covered with TFM tissue freezing medium and frozen on a metal block cooled with liquid nitrogen. They were stored long-term at −80 °C. Cryostat sections of 12–25 micron thickness were placed on slides and stored at −20 °C. Sections were processed for in situ hybridization using an RNAscope^®^ probe for mouse *Axl* with the RNAscope^®^ 2.5 HD Assay-Red kit following the instructions of the manufacturer (ACD). This included a boiling step and protease treatment for antigen retrieval before adding the probe. Positive signals are pink/magenta dots when viewed with brightfield or bright red when viewed using a fluorescence microscope. Some slides were counterstained with 50% Gill’s Hemotoxylin I. Other slides were post-processed for immunofluorescent co-labeling of hair cells by treating with blocking solution of 5% horse serum in PBS and incubating with rabbit anti-myosinVI as the primary antibody and donkey anti-rabbit AlexaFluor 488 as the secondary antibody.

### 2.5. Microscopy and Evaluating Infection

Samples were screened and photographed with a Spot CMOS camera attached to a Nikon Eclipse E800 microscope. Selected samples were imaged using 10X 20X and/or 60X objectives with a Nikon confocal microscope (Purdue University) or a Zeiss LSM800 confocal microscope (The Jackson Labroatory). Typically, the entire cochlea was reconstructed from Z-stacks taken at a lower power, and then 3–6 Z-stacks were taken with the 60X oil-immersion objective, roughly equally spaced along the length of the cochlea from base to apex. All Z-projections were created using the maximum intensity algorithm in NIH ImageJ. Images were processed and analyzed with NIH ImageJ and any additional image enhancement (brightness increase) was done with Adobe Photoshop and is indicated in the figure legend.

Analysis of cell infection with ZIKV was conducted via a careful evaluation of confocal image stacks taken with a 60X lens. After merging the colors for each antibody label into single slices of the Z-stack, we scrolled through the Z-stack to detect dsRNA-positive pixels. An obvious cluster of positive pixels was used to indicate infected cell(s). To determine that a hair cell was indeed infected, and not simply surrounded by the processes of infected supporting cells, we required overlap of anti-dsRNA and anti-myosinVI signals (the latter distributed throughout the hair cell cytoplasm). To determine that supporting cells were infected with ZIKV, we looked for clustered dsRNA-positive pixels within the supporting cell layer; this layer was marked by dense, small nuclei labeled with an anti-Sox2 antibody to the transcription factor SOX2. Clustered foci of anti-dsRNA in the supporting cell layer were considered evidence of infection of this cell type, because no other cells occupy this layer. Similar criteria were used to judge ZIKV infection of cells within the GER, a domain that was also Sox2-positive. Mesenchymal cells were judged to be infected when we saw larger clusters of dsRNA-positive pixels located beneath the epithelial layers of the cochlear duct. These judgements of positive infection were always made by comparison to control (uninfected) cochleae from the same experimental batch, which were fixed and immunolabeled in parallel and imaged within a few days of each other. The control cultures were usually devoid of larger anti-dsRNA puncta that marks viral replication. Some control batches showed diffuse speckled labeling of the surface of the culture, but this was readily distinguished from a more clustered distribution of a larger puncta characteristic of cellular infection. The former was classified as a staining artifact.

### 2.6. Image Analysis of Cell Death

Confocal image stacks, captured with a 60X objective, were prepared from the base, middle and apical parts of each cochlea. In each stack, we selected a sample depth of 20 slices to include the Sox2-positive supporting cell region. This was collapsed into a maximum intensity Z-projection. Separate region of interest (ROI) outlines were made for the organ of Corti and the GER, which can be recognized by differences in the size and density of the Sox2-labeled cell nuclei. These outlines were saved in the ROI manager for subsequent use. Each Z-projection was then split into three color channels, to isolate the active Caspase-3 (red) signal. The red channel was despeckled three times and auto-thresholded using the “Max Entropy” algorithm with the threshold set to 50–255. This was empirically judged to most closely resemble the clumped caspase signals observed in the original Z-projection, while discarding background pixels. These images were quantified in two ways. In the first, we calculated the proportion of each sample area that was occupied by caspase-positive pixels. A macro was created and run to generate area measurements for the ROIs and to quantify the caspase-positive areas within each ROI as a fraction of the total ROI area. In the second method, a macro was created to count the number of separate caspase foci in each ROI, to approximate the number of caspase-positive cells. For both methods, the values from individual stacks from the same cochlea were averaged. Neither method revealed significant differences between the controls and ZIKV cochleae, with separate statistical comparisons done for the organ of Corti and the GER (Graphpad Prism 5 software, one-tailed t-test, all *p* values > 0.1).

## 3. Results

### 3.1. ZIKV Infects Both Prosensory (Mitotic) Cells and Post-Mitotic Sensory Cells in Cochlear Organ Cultures

**Figure 1:**
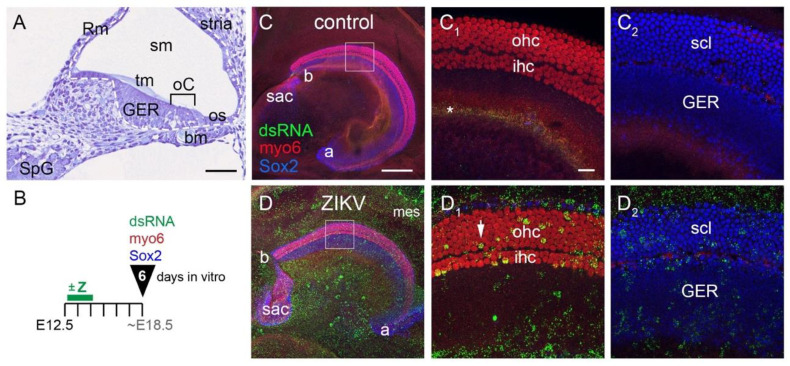
Cochlear architecture shown in situ on E18.5 and after E12.5 explants were cultured for 6 days. (**A**) A histological section through an E18.5 cochlea stained with hematoxylin and eosin. The epithelium of the cochlear duct is outlined with a dashed white line. The floor of the cochlear duct includes cells of the organ of Corti (oC) and the greater epithelial ridge (GER). In this turn of the cochlea, medial is towards the left and lateral is towards the right. Scale bar = 100 microns. (**B**) Experimental design for panels **C** and **D** depicting explants prepared on E12.5, with a green bar for a 48 h window, beginning 4–6 h after the culture is initiated, to indicate with or without the addition of ZIKV ( +Z). After fixation (black arrowhead) at 6 DIV, triple immunostaining was done with dsRNA, myo6 and Sox2 antibodies. (**C**,**D**) Maximum-intensity Z-projections of cochleae immunolabeled as indicated. Asterisk in C_1_ denotes an area of background dsRNA labeling on the surface of the tissue. High power views of the boxed areas are shown in the adjacent two panels, focused on either the hair cell layer (**C_1_**,**D_1_**) or the supporting cell layer (**C_2_**,**D_2_**) by including only 10 consecutive optical slices. White arrowhead depicts a dsRNA-positive outer hair cell. Scale bars = 200 microns for low power images (panels **C**,**D**) and 20 microns for high power images. Abbreviations: a, apex of cochlea; b, base of cochlea; bm, basilar membrane; E, embryonic day; GER, greater epithelial ridge; ihc, inner hair cells; mes, mesenchyme; oC, organ of Corti; ohc, outer hair cells; os, outer sulcus; Rm, Reissner’s membrane; sac, saccular macula; scl, supporting cell layer; sm, scala media; SpG, spiral ganglion; stria, stria vascularis; tm, tectorial membrane; Z, ZIKV.

The mouse cochlea undergoes major morphogenetic and cellular changes that could affect its susceptibility to ZIKV as development progresses. To assess this possibility, we isolated cochleae from mouse embryos at three key time points (E12.5, E15.5 and P2) that span from the proliferative to the mid-differentiation stages of sensory hair cells. To orient the reader to cochlear morphology at a time equivalent to E12.5 plus 6 DIV, we show a cross-section through a cochlear duct fixed and stained in situ at E18.5 (Figure 1A). The organ of Corti is a narrow domain present on the floor of the cochlear duct and is occupied by inner and outer hair cells and their underlying supporting cells. The acellular tympanic membrane spans across the sensory epithelium. The organ of Corti is flanked by the GER on one side and the outer sulcus on the other side (located towards the outside of the cochlear spiral). Mesenchymal cells of the basilar membrane reside beneath the organ of Corti. The epithelial cells derived from the roof of the cochlear duct will associate with adjacent cells to form two specialized structures: Reissner’s membrane and the stria vascularis.

Explants were cultured and treated with ZIKV as described in Materials and Methods. After 6 DIV, infected cells were detected using an antibody directed against dsRNA. Young hair cells express myosin-6, and both supporting cells of the organ of Corti as well as the cells of the GER express SOX2. Addition of ZIKV at all three ages resulted in robust infection of the cochleae and the surrounding mesenchyme. As a general observation, ZIKV infection was evident within each of the major cell types and domains of the epithelium, as compared to controls from the same experiment that were processed in parallel. Exemplar specimens are shown for cochlear cultures established from an E12.5 mouse and cultured for 6 DIV (Figure 1B). The control cochlea (Figure 1C) has only background labeling with the dsRNA antibody. For the ZIKV specimen (Figure 1D), virus was washed out with a media change 48 h after it was added to the culture. Infected hair cells were defined by having large dsRNA-positive puncta that overlapped with myosin-6 immunoreactivity in optical slices captured with a 60X lens. See Methods Section 2.5 for further criteria used to identify virus-infected cells of different types. Using established criteria, virus was localized to cells throughout the cochlear epithelium and the surrounding tissues (both epithelial and mesenchymal). Mesenchymal cells were often the most intensely immunoreactive for dsRNA and were readily apparent at low power (Figure 1D). When included in the cultures, the epithelial roof of the cochlear duct and its associated mesenchyme were also found to be infected. Within the cochlear duct, infected cells were observed in the GER. ZIKV dsRNA was detected in both inner and outer hair cells and in the SOX2-postive supporting layer located beneath the hair cells. Confocal image slices taken at the level of the hair cells (Figure 1(D_1_)) or at the level of the supporting cells and the GER (Figure 1(D_2_)) allow for separate confirmation of dsRNA within these cell types.

#### 3.1.1. E12.5 Plus 6 Days In Vitro

One purpose of this study was to assess the susceptibility of cochlear cells to viral infection at different developmental stages. To narrow the time window of potential infection, we reduced the duration of ZIKV addition to 24 h, beginning on the day the explant was placed in vitro. Mouse cochlear cultures were initiated on E12.5. At this time, the cochlear duct is lined with an actively mitotic epithelium [19]. At a histological level in situ, the E12.5 cochlear epithelium is thicker on the floor of the duct (the prosensory side) and thinner on the roof of the duct that will develop into specific non-sensory cochlear tissues. The duct epithelium rests on a basal lamina and is surrounded by mesenchymal cells. These mesenchymal were included in the cultures.

**Figure 2:**
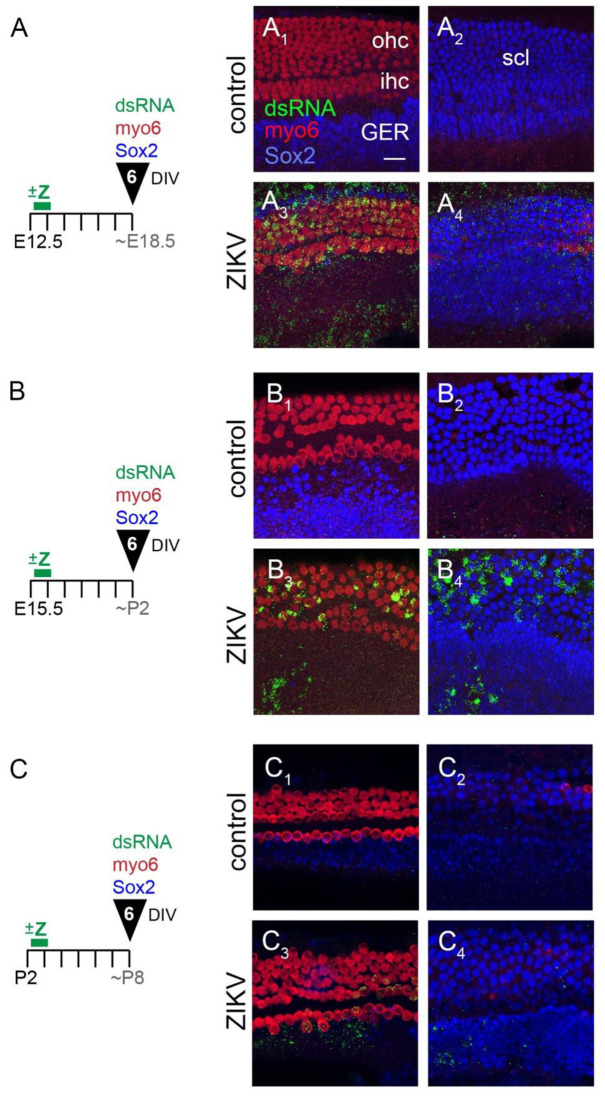
Cochlear cultures were infected with ZIKV at (**A**) E12.5, (**B**) E15.5 and (**C**) P2 and processed after 6 DIV. Images are Z-projections from 10 adjacent optical slices through either the hair cell layer (**left columns**) or the supporting cell layers (**right columns**). The myo6 and Sox2 signals for panel (**C**) were brightened to improve the contrast. Scale bar = 20 microns. Abbreviation: DIV, days in vitro; P, postnatal day; all other abbreviations as in Figure 1 legend.

ZIKV was added to the E12.5 cultures after 4–6 h in vitro, washed out 24 h later, and the cells were allowed to differentiate for a total of 6 DIV, as indicated schematically (Figure 2A). Controls lacked the puncta of dsRNA labeling that characterized infected cells (Figure 2(A_1_,A_2_)). The same cell types (hair cells, supporting cells, GER and mesenchyme) labeled for dsRNA after this shorter ZIKV incubation time (Figure 2(A_3_,A_4_)), as was observed with a 48 h incubation. This figure is representative of 10 cochleae infected with ZIKV across 3 independent experiments, with 9 control explants processed in parallel.

#### 3.1.2. E15.5 Plus 6 Days In Vitro

In the intact animal, the prosensory domain of the mouse begins to pull out of division between E13 and E14, with the apex exiting the cell cycle slightly in advance of the base [19]. By E14, over 80% of the cells in the future organ of Corti are no longer dividing, which demarcates a so-called “zone of non-proliferation” [20]. The cessation of proliferation in the organ of Corti primordium is under the control of the cyclin-dependent kinase inhibitor 1b, Cdkn1b (also known as p27^Kip1^) [20]. Most of the remaining cells of the duct floor continue to proliferate for several more days. At the time E15.5 cultures were initiated and infected with ZIKV, the zone of non-proliferation that marks the future organ of Corti is well established. Thus, this time point was chosen to determine whether post-mitotic sensory cells are susceptible to ZIKV infection. Four independent experiments were conducted, with a total of 38 organs (20 controls and 18 ZIKV) analyzed. Similar to Section 3.1.1, virus was added on the first day of culture and washed out after 24 h (Figure 2B). When processed after 6 DIV, the control cultures were devoid of punctate labeling with anti-dsRNA (Figure 2(B_1_,B_2_)). In total, all but one of the 18 organs exposed to ZIKV displayed well-infected hair cells (Figure 2(B_3_)). Nearly all of these ZIKV-treated specimens also showed infection in the supporting cell layer and in the GER (Figure 2(B_4_)).

#### 3.1.3. P2 Plus 6 Days In Vitro

Cultures initiated at P2 have already established a single row of inner hair cells and 3–4 rows of outer hair cells throughout the apical-basal longitudinal axis of the cochlea. All hair cells and supporting cells are post-mitotic at this time [19]. ZIKV was added for a 24 h window at the start of these cultures. The experimental conditions for P2 plus 6 DIV are shown in Figure 2C. Controls showed little to no dsRNA labeling (Figure 2(C_1_,C_2_)). The addition of ZIKV showed that postnatal hair cells, supporting cells and GER cells are still susceptible to infection (Figure 2(C_3_,C_4_)). The amount of infection appears lower than for the embryonic explants, both in the amount of dsRNA-positive signal per infected cell and in the number of infected cells. This is apparent for both hair cells and supporting cells.

In summary, the cells of the organ of Corti, the GER and the adjacent tissues are susceptible to ZIKV at E12.5, E15.5 and P2. No consistent phenotypic abnormalities were present in infected cells, and the organ’s overall morphology was not obviously different between controls and ZIKV-treated samples at any of the ages tested.

### 3.2. ZIKV Infection Does Not Induce Extensive Cell Death in the Cochlear Epithelium

Cochlear cultures actively infected with ZIKV had normal tissue morphology up to 6 days later. Swollen cells characteristic of necrosis or cell fragments associated with apoptosis were observed rather infrequently. Because ZIKV can lead to increases in cell death in other cell types, including both embryonic and adult neural progenitors [11,21,22], we evaluated infected cultures for possible changes in cell death using an antibody to active Caspase-3. Evaluation of each cochlea was done by creating confocal image stacks using a 60X objective and deliberately sampling from 3–5 regions along the apical-basal length of the cochlea. Three conditions were evaluated: E12.5 plus 3 DIV (2 controls, 4 ZIKV), E15.5 plus 6 DIV (2 controls, 3 ZIKV) and E15.5 plus 3 DIV (16 controls, 15 ZIV).

**Figure 3:**
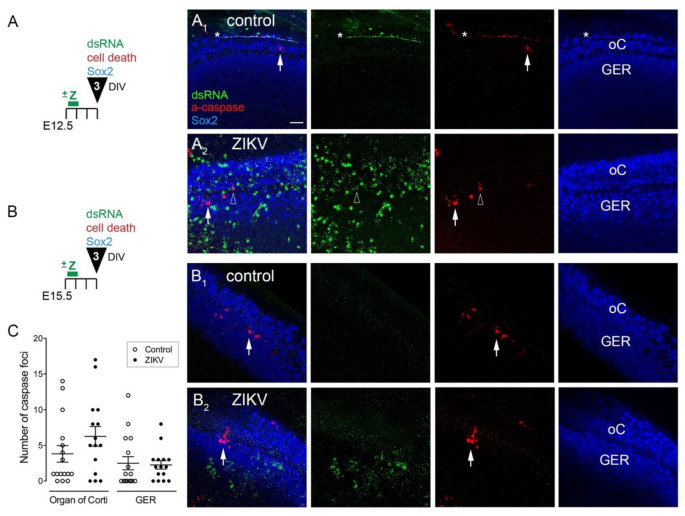
ZIKV infection does not induce extensive cell death in cochlear cultures. (**A**,**B**) Schematics of the experimental design for organs explanted on either E12.5 (**A**) or E15.5 (**B**), where ZIKV (Z) was added to experimental but not control cultures on the first day in vitro. Specimens were fixed after 3 days in vitro. (**A_1_**,**A_2_**,**B_1_**,**B_2_**) Confocal image stacks (Z-projections from 20 slices) taken through the Sox2-positive layers (supporting cells and the GER). The color channels are indicated in panel A and are shown superimposed (**column 1**) or separated (**columns 2**–**4**). White arrows point to foci positive for active Caspase-3. Open arrowhead depicts a rare cluster that is double-positive for active Caspase-3 and dsRNA. The asterisks denote a line of presumed background fluorescence on the edge of the organ, where signals were detected in all three color channels. Scale bar = 20 microns. (**C**) Quantification of active Caspase-3-positive foci from image stacks of E15.5 +3 DIV cochleae; each dot represents an average value from several image stacks taken through an individual cochlea. There is no statistical difference between controls and ZIKV conditions for either the organ of Corti or the GER (one-tailed t-test, *p* > 0.1). Abbreviations: a-caspase, active Caspase-3; DIV, days in vitro; all other abbreviations as in Figure 1 legend.

Z-projections of images from a pair of cochleae are shown from the E12.5 plus 3 DIV experiment (Figure 3A). The control (untreated with ZIKV) showed caspase labeling in the organ of Corti (Figure 3(A_1_), arrow). Figure 3(A_2_) presents an exceptionally well-infected ZIKV sample that demonstrates three findings: (1) the overwhelming majority of dsRNA-positive cells did not co-label with active Caspase-3; (2) caspase-positive cells were relatively sparse in the cochlea and typically were dsRNA-negative; and (3) on rare occasions we observed cells double-positive for dsRNA and active Caspase-3 (Figure 3(A_2_), open arrowhead). This image stack has caspase-positive cells in both the organ of Corti and in the GER.

The E15.5 plus 3 DIV experiments were quantified for active Caspase-3 foci, with separate analysis done on the GER and the organ of Corti. SOX2 immunolabeling was used to locate these regions. (Figure 3B). Both control and ZIKV explants were observed to have a few caspase-positive foci in the sampled regions (Figure 3(B_1_,B_2_)). The number of such foci per image stack was not statistically different between controls and ZIKV samples (Figure 3C). It should be noted that these experiments were conducted with a virus stock whose titer had dropped almost 10-fold over time, such that only 50% (*n* = 26/53) of the regions selected to image had dsRNA-positive cells within the SOX2 expression domains. It is possible that the results may have been different if we were able to obtain higher levels of infection. Despite this caveat, there was limited spatial overlap between cells expressing dsRNA (actively ZIKV-infected) and cells labeled for active Caspase-3. Thus, we found no evidence for an increase in cell death in cochleae after 3 days of culture in the presence of ZIKV.

### 3.3. ZIKV Infection of the Cochlea Is Blocked by Preincubation with a Neutralizing Antibody

**Figure 4:**
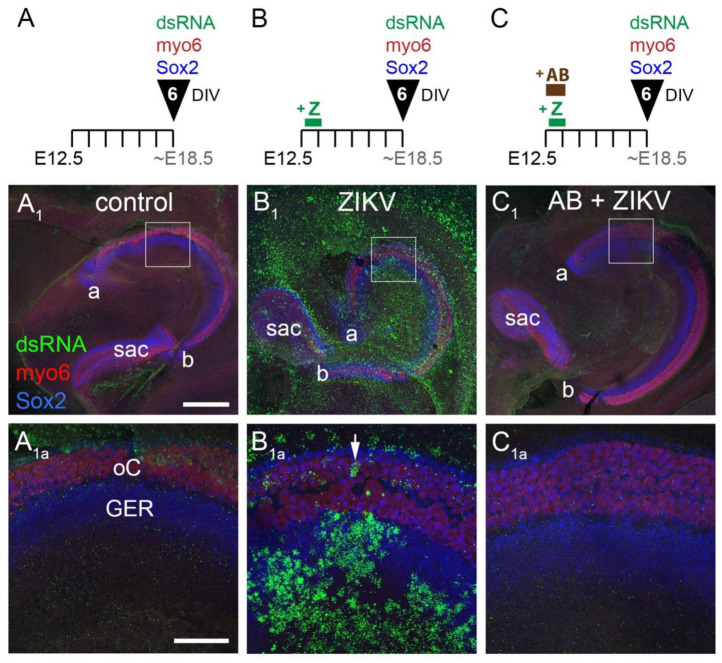
ZIKV infection of E12.5 cochlear explants is effectively neutralized by the ZIKV-117 antibody. Top row shows the experimental conditions, with exemplar cochleae for each condition shown at low power in the middle row. The regions outlined by white boxes are shown at higher power in the panels of the bottom row. The high-power images are Z-projections combined across 58 optical slices to include both hair cell and supporting cell layers. White arrow points to an infected hair cell. Scale bars = 200 microns for low power (middle row) and 50 microns for high power (bottom row). Abbreviations: DIV, days in vitro. All other abbreviations see Figure 1 legend.

A particularly potent neutralizing antibody is ZIKV-117, whose binding to the ZIKV surface has been studied using high-resolution structural biology approaches [23,24]. The presence of the antibody can block the virus from infecting host cells in vitro [12]. We tested this effect on mouse cochlear explants. Three conditions were tested in cultures established at E12.5. In the first condition (control, *n* = 7), explants were untreated (Figure 4A). These showed very little signal for dsRNA (Figure 4(A_1_,A_1a_)). In the second condition (ZIKV, *n* = 6), explants were given 24 h of exposure to ZIKV and then given a media change (Figure 4B). These were labeled with dsRNA (Figure 4(B_1_,B_1a_), *n* = 6). In the third condition (AB + ZIKV, *n* = 6), explants were treated with ZIKV-117 antibody for 6 h to allow for penetration into the duct, and then ZIKV was added. A media change 24 h later was used to remove the antibody and the virus (Figure 4C). All explants were allowed to develop for a total of 6 DIV. Processing the tissue to detect dsRNA and cell types showed almost a total block of ZIKV infection when added to the cultures in the presence of the neutralizing antibody (Figure 4(C_1_,C_1a_)).

### 3.4. Axl Transcripts Are Expressed in Many Tissues of the Developing Inner Ear

The cochlear tissue tropism for ZIKV infectivity was quite ubiquitous at the stages examined, suggesting that the host cell receptor(s) used by the virus to gain entry into these cells is likewise broadly distributed within the cochlea. Although the full range of cellular receptors and attachment factors used by ZIKV is still being investigated, there is evidence that the surface protein AXL promotes ZIKV entry into mammalian cells using both gain-of-function and loss-of-function approaches [25,26,27,28,29]. On the other hand, it is not strictly required for ZIKV susceptibility (reviewed by [16]). AXL is a member of the TAM family of receptor tyrosine kinases. TAM receptors bind secreted ligands to sense phosphotidylserine on membranes of apoptotic cells and facilitate their clearance. TAMs also play a key role in attenuating innate immunity after an inflammatory response to pathogens has been activated (reviewed by [30]).

Even if there is redundancy among ZIKV receptors/attachment factors, it can still be useful to map their expression in permissive organs, such as the cochlea. RNA-seq experiments indicate that transcripts encoding all 3 TAM proteins (TYRO3, AXL and MER) are expressed in embryonic and neonatal cochleae. They are differentially expressed in hair cells and supporting cells, and their levels vary with the stage of development (see www.umgear.org, accessed 15 July 2021 [31,32,33,34]). However, these methods lack spatial and temporal resolution.

**Figure 5:**
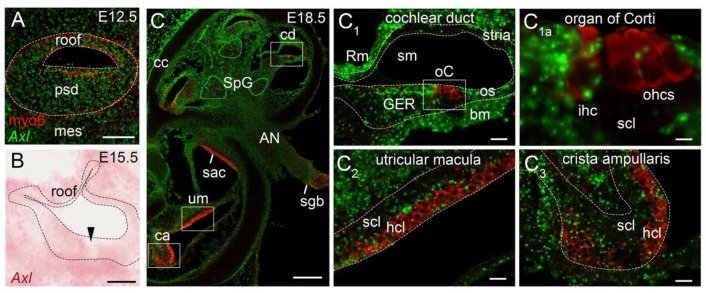
*Axl* is expressed in the embryonic mouse cochlea. RNAscope^®^ probes were detected in cryostat sections and imaged via either confocal (panels **A**,**C**) or brightfield (panel **B**) microscopy. Within the inner ear, dashed lines indicate the epithelial layers. Double-labeling of *Axl* (green) and anti-myosin-6 (red) is shown in (panels **A**,**C**); in these confocal images, pseudocoloring of *Axl* as a green signal offered better clarity. All higher power images of the cochlear duct are shown with the center of the cochlear spiral (called medial) to the left. (**A**) The E12.5 cochlear duct is shown as a maximum-intensity Z-projection of 23 confocal image slices (approximately 20 microns of depth). By enhancing the contrast, *Axl* expression can be observed throughout the thick prosensory domain (psd), the thin non-sensory roof and the surrounding mesenchyme (mes). Scale bar = 50 microns. (**B**) At E15.5, *Axl* (pink) is expressed in the cochlear duct, but with an obvious gap of reduced *Axl* expression (arrowhead) that was reproducibly observed midway along the floor of the duct. The roof has partly collapsed onto itself. Scale bar = 50 microns. (**C**) Low power view of a single confocal slice through the E18.5 inner ear with the cochlea in the top half and 3 vestibular organs in the bottom half. Cross-sections through the spiral ganglion are outlined in solid green. The auditory nerve (AN) exits the cochlea and will enter the brain (removed at dissection) at the Schwann-glial border (sgb). Single confocal slices of the boxed areas are shown at higher power. *Axl* (green) is present in nearly all of the epithelial and mesenchymal tissues of the cochlea, except as noted in the text. At least some hair cells and supporting cells appear to express *Axl* in all inner ear sensory organs. Hair cell expression of *Axl* is shown at higher power in (panel **C_1a_**). Scale bars: **C** = 200 microns; **C_1_**, **C_2_**, **C_3_** = 20 microns; **C_1a_** = 5 microns. Abbreviations: AN, auditory nerve; bm, basilar membrane; ca, crista ampullaris; cc, cochlear capsule; cd, cochlear duct; GER, greater epithelial ridge; hcl, hair cell layer; ihc, inner hair cell; mes, mesenchyme; oC, organ of Corti; ohcs, outer hair cells; os, outer sulcus; psd, prosensory domain; Rm, Reissner’s membrane; sac, saccular macula; scl, supporting cell layer; sgb, Schwann-glial border; sm, scala media; SpG, spiral ganglion; stria, stria vascularis; um, utricular macula.

In this study, we examined the distribution of *Axl* transcripts in the developing mouse cochlea at the histological level at 6 time points (E12.5, E13.5, E15.5, E17.5, E18.5, P2) by sampling 2–5 embryos per age. This spans the time window of the culture experiments. In situ hybridization on cryostat sections of mouse heads was used to localize *Axl* at stages when ZIKV readily infects the cochlea in vitro. The earliest timepoint examined was E12.5, which showed broad but weak *Axl* labeling throughout the epithelium of the cochlear duct, including both its thicker prosensory domain and the thinner non-sensory roof of the duct (Figure 5A). The mesenchyme surrounding the inner ear also expressed *Axl*, whereas expression in the spiral ganglion was scattered and even weaker by comparison (data not shown). At E13.5, *Axl* signal intensity in the cochlea remained weak and generally resembled E12.5 in its tissue distribution (data not shown).

By E15.5, the floor of the cochlear duct showed a reproducible gap in *Axl* expression (Figure 5B, black arrowhead). This gap was associated with the position of the developing organ of Corti, as indicated by double-staining with antibody to myosin-6 (shown at E18.5 in Figure 5(C_1_)). Both inner and outer hair cells and their associated supporting cells were located within the gap. We used confocal imaging of E18.5 specimens to address whether or not individual hair cells of the organ of Corti retained any *Axl* signal (Figure 5C), since the cell culture data showed they were still susceptible to ZIKV beyond this age. Indeed, selected examples of both hair cell types presented with spatial overlap of *Axl*-positive puncta and the cytoplasmic marker, myosin-6. This was more frequently observed for inner hair cells (Figure 5(C_1a_)). In contrast, some inner hair cells and the majority of outer hair cells did not appear to have detectable levels of *Axl* transcripts. Turning our attention to the supporting cell layer located beneath the hair cells, we observed a scattering of *Axl*-positive puncta here. The single row of supporting cells associated with inner hair cells that will mature into inner phalangeal cells were reliably *Axl*-positive. Nonetheless, the observation of lower levels of *Axl* specifically within the organ of Corti was evident in all specimens examined from E15.5 through P2.

The organ of Corti can be contrasted with higher *Axl* expression in the epithelial cells of the GER that flank the organ on its medial side and the outer sulcus that flank its lateral side (Figure 5(C_1_)). Most cells of the GER and the outer sulcus become post-mitotic 2–4 days after the organ of Corti in the mouse [19]. A large fraction of GER cells will disappear by postnatal day 14 through autophagy [35] and apoptosis [36].

The spiral ganglion, where the cell bodies of the primary sensory neurons of the cochlea are located, appears to have an intermediate level of *Axl* expression (Figure 5C) when compared to the otic capsule (negative) or the strong positive signals in the cochlear mesenchyme within the cochlea. Strong mesenchymal signals include the tissue in the center of the cochlea (Figure 5C) and the cells beneath the epithelium on the roof (Reissner’s membrane anlage), the basilar membrane and the lateral wall (stria vascularis anlage) of the cochlear duct (Figure 5(C_1_)).

A high level of *Axl* expression at E18.5 was observed in cells dispersed within the auditory nerve as it travels through the cochlea (Figure 5C). We presume *Axl* was expressed by Schwann cells and/or fibroblasts in the peripheral part of this nerve, because there was an abrupt decrease in *Axl* levels precisely where the auditory nerve entered the central nervous system at the Schwann-glial border (Figure 5C). Here the Schwann cells and fibroblasts give way to oligodendrocytes and astrocytes as the auditory nerve enters the brain. In contrast to these examples, the cartilage cells of the otic capsule are consistently low in *Axl* expression (Figure 5C). The hair cell and supporting cell layers of the vestibular organs (utricular macula, saccular macula, crista ampullaris) showed scattered *Axl* expression on E18.5 (Figure 5(C_2_,C_3_)); we did not examine the vestibular organs at P2.

## 4. Discussion

In this study, cochlear explants derived from Swiss Webster mice were used to evaluate ZIKV tropism for the sensory organ of hearing. Unlike in vivo mouse studies which required genetic strains with compromised interferon signaling to evade the immune response, we show that cochlear explants were susceptible to ZIKV in a wild-type strain. In a recent study, ZIKV readily infected inner ear tissues lacking a type I interferon receptor following a systemic route of virus delivery (foot pad injections) in adult *ifnar1^−/−^* mice [10]. Our study thus fills in a developmental gap, revealing ZIKV susceptibility at a mitotic stage of organogenesis and extending that to the early postnatal period, when the organ of Corti is post-mitotic. These stages approximately correspond to the first 4 months of human embryogenesis. In humans, the prosensory cochlea is postmitotic at 10 weeks of gestation, hair cells begin to appear at 12 weeks and the full complement of hair cells is present in the middle turn at 15 weeks [37,38].

The penetrance of hearing loss associated with Congenital ZIKV Syndrome varies widely across studies (reviewed by [4]). One retrospective study that examined 69 infants presenting with microcephaly indicated a 6% penetrance within the first 10 months after birth using ABR as a screening tool [39]. Overall, studies using ABR screens detected hearing loss in 0–29% of infants with presumed or verified ZIKV exposure in utero [4]. Using OAE as a screening tool, ZIKV exposure correlated with hearing loss in 0–75% of infants [4]. It remains to be determined why there is such high variability in penetrance of hearing loss between studies, although this could result from differences in inclusion criteria of the subjects. High variability of ZIKV infection in the brain and inner ear of chicken embryos was evident in previous studies from our laboratory [9,40]. Similarly, we observed considerable differences in the overall amount of ZIKV infection across samples in this study, even when comparing within a single experiment. It is not known how much of this variability might be due to experimental conditions (such as virus draining away from some explants). Despite this variability, overall, we have confirmed that direct ZIKV infection of the embryonic mammalian hearing organ is possible and might underlie at least some of the hearing deficits found in human infants exposed to ZIKV in utero.

One unexpected finding was that ZIKV infection did not significantly increase cell death in either the organ of Corti or the GER. In the central nervous system of chicken embryos, regions with high levels of ZIKV infection were shown to overlap with excessive levels of cell death within 3 days of exposure to the virus in ovo [40]. Likewise, in this same species, infection of the embryonic statoacoustic ganglion of the inner ear caused a marked shrinkage of this structure, which we speculate was caused by increased cell death observed a few days earlier [9]. On the other hand, infected sensory organs of the embryonic chicken inner ear appeared resistant to the pathological effects of ZIKV. Results differed in the *Ifnar1^−/−^* mouse cochlea infected with ZIKV at 5–6 weeks of age. In this model, ZIKV infection led to cellular pathologies in the inner ear 9 days after systemic delivery of the virus [10]. Readouts of the cell death effectors such as active Caspase-3 were not evaluated, although other cellular proteins associated with stress were abnormally increased. In the current study, neither quantitative analysis of active Caspase-3 after 3 days nor morphological assessment after 6 days revealed pathological changes in the cochlear epithelium due to ZIKV infection. Since we did not include physiological assessment of organ explants, we have no information on possible functional consequences of an ongoing ZIKV infection in vitro. Perhaps a longer survival time is needed for pathologies to manifest, or perhaps the embryonic ages and/or genetic backgrounds may explain the difference in outcome for this study, when compared to that of *Ifnar1^−/−^* adult mice [10]. Alternatively, the embryonic hearing organ may be able to harbor ZIKV without detrimental consequences. If this finding could be confirmed and extended to humans, it may help to explain why hearing loss associated with inner ear pathophysiology, as indicated by reduced otoacoustic emissions, is a comorbidity for only a minority of patients with congenital ZIKV syndrome [39].

Our results showed that addition of a well-characterized neutralizing antibody [12] that binds to the envelope proteins of ZIKV [24] was effective in blocking ZIKV from establishing a productive infection of cochlear explants. Presumably, the antibody interfered with the binding of ZIKV to the host cell receptor(s). The delivery of mRNA encoding this monoclonal antibody is currently being explored as a protective treatment against ZIKV infection, with initial experiments conducted in mice [14].

We examined the spatiotemporal pattern of *Axl* expression in the developing cochlea to determine whether its distribution overlapped with ZIKV susceptibility. We initiated this line of inquiry because of data showing that AXL can promote the entry and/or replication of flaviviruses in other tissues and organs (reviewed by [16]). AXL has at least two modes of action to enhance flavivirus infection of cultured cells: (1) its extracellular domain facilitates virus attachment using the GAS6 ligand as a bridge between the host cell and the virus membrane and (2) its intracellular kinase activity boosts viral production by muting type I interferon signaling to reduce the cellular antiviral response [27,29,41]. *Axl* expression correlates with permissiveness to ZIKV in many human host cells, including skin cells [28], neural progenitors and embryonic astrocytes and microglia [26,27]. However, AXL is unlikely to be the sole attachment factor for ZIKV based on results from AXL deficiency in mammalian cell lines [29,42]. A similar conclusion was reached for mice in vivo, because brains, eyes and testes retained their susceptibility to ZIKV infection in AXL-deficient mice [25,43,44,45]. Likewise, ZIKV-mediated pathology was not prevented in neonatal *Axl*-knockout mice [45]. Moreover, a positive correlation between ZIKV permissivity and *Axl* expression in the brains of newborn mice is evident in only some, but not all, brain regions [45].

The ubiquitous expression of *Axl* in the E12.5 embryonic cochlea overlaps with a broad tropism observed for ZIKV at this age, but this correspondence weakens as the sensory primordium pulls out of division between E12.5 and E14.5. By E15.5 and continuing into the early postnatal period, *Axl* transcripts are significantly reduced in both hair cells and supporting cells of the developing organ of Corti, and yet, these cells retain at least moderate susceptibility to infection by ZIKV delivered to cochlear explants. This suggests that for the cells of the organ of Corti, like many other tissues in the mouse, AXL may not be an exclusive cell surface protein mediating ZIKV attachment.

The global threat to human health posed by pathogenic flaviviruses is likely to continue for the foreseeable future [46], necessitating further progress in understanding virus-induced pathologies. ZIKV now joins several pathogens whose exposure to the gestating fetus correlates with congenital hearing loss after birth [47,48]. Knowing whether infection of the peripheral auditory system in utero could underlie some aspects of the ensuing hearing loss is necessary for choosing appropriate therapeutic treatments. One strategy to reveal viral tropism in relatively inaccessible tissues with complex cytoarchitectures, such as the embryonic inner ear, is demonstrated in this study through the use of organ cultures in animal models.

## Supplementary Materials

The following are available online at https://www.mdpi.com/article/10.3390/v13091823/s1, Table S1: Summary of experimental conditions for cochlear cultures.

## References

[1] Brasil P., Pereira J.P., Moreira M.E., Ribeiro Nogueira R.M., Damasceno L., Wakimoto M., Rabello R.S., Valderramos S.G., Halai U.A., Salles T.S. (2016). Zika Virus Infection in Pregnant Women in Rio de Janeiro. N. Engl. J. Med..

[2] Moore C.A., Staples J.E., Dobyns W.B., Pessoa A., Ventura C.V., Fonseca E.B., Ribeiro E.M., Ventura L.O., Neto N.N., Arena J.F. (2017). Characterizing the Pattern of Anomalies in Congenital Zika Syndrome for Pediatric Clinicians. JAMA Pediatr..

[3] Nielsen-Saines K., Brasil P., Kerin T., Vasconcelos Z., Gabaglia C.R., Damasceno L., Pone M., de Abreu Carvalho L.M., Pone S.M., Zin A.A. (2019). Delayed childhood neurodevelopment and neurosensory alterations in the second year of life in a prospective cohort of ZIKV-exposed children. Nat. Med..

[4] Barbosa M.H.M., Magalhaes-Barbosa M.C., Robaina J.R., Prata-Barbosa A., Lima M., Cunha A. (2019). Auditory findings associated with Zika virus infection: An integrative review. Braz. J. Otorhinolaryngol..

[5] Escobar-Ipuz F.A., Soria-Bretones C., Garcia-Jimenez M.A., Cueto E.M., Torres Aranda A.M., Sotos J.M. (2019). Early detection of neonatal hearing loss by otoacoustic emissions and auditory brainstem response over 10 years of experience. Int. J. Pediatr. Otorhinolaryngol..

[6] Thompson D.C., McPhillips H., Davis R.L., Lieu T.L., Homer C.J., Helfand M. (2001). Universal newborn hearing screening: Summary of evidence. JAMA.

[7] Bakhos D., Marx M., Villeneuve A., Lescanne E., Kim S., Robier A. (2017). Electrophysiological exploration of hearing. Eur. Ann. Otorhinolaryngol. Head Neck Dis..

[8] Guinan J.J., Salt A., Cheatham M.A. (2012). Progress in cochlear physiology after Bekesy. Hear Res..

[9] Thawani A., Sammudin N.H., Reygaerts H.S., Wozniak A.N., Munnamalai V., Kuhn R.J., Fekete D.M. (2020). Zika virus can directly infect and damage the auditory and vestibular components of the embryonic chicken inner ear. Dev. Dyn..

[10] Yee K.T., Neupane B., Bai F., Vetter D.E. (2020). Zika virus infection causes widespread damage to the inner ear. Hear Res..

[11] Tang H., Hammack C., Ogden S.C., Wen Z., Qian X., Li Y., Yao B., Shin J., Zhang F., Lee E.M. (2016). Zika Virus Infects Human Cortical Neural Progenitors and Attenuates Their Growth. Cell Stem Cell.

[12] Sapparapu G., Fernandez E., Kose N., Bin C., Fox J.M., Bombardi R.G., Zhao H., Nelson C.A., Bryan A.L., Barnes T. (2016). Neutralizing human antibodies prevent Zika virus replication and fetal disease in mice. Nature.

[13] Almansour I., Alfares R., Aljofi H. (2018). Large-scale analysis of B-cell epitopes of envelope: Implications for Zika vaccine and immunotherapeutic development. F1000Research.

[14] Erasmus J.H., Archer J., Fuerte-Stone J., Khandhar A.P., Voigt E., Granger B., Bombardi R.G., Govero J., Tan Q., Durnell L.A. (2020). Intramuscular Delivery of Replicon RNA Encoding ZIKV-117 Human Monoclonal Antibody Protects against Zika Virus Infection. Mol. Ther. Methods Clin. Dev..

[15] Robbiani D.F., Bozzacco L., Keeffe J.R., Khouri R., Olsen P.C., Gazumyan A., Schaefer-Babajew D., Avila-Rios S., Nogueira L., Patel R. (2017). Recurrent Potent Human Neutralizing Antibodies to Zika Virus in Brazil and Mexico. Cell.

[16] Xie S., Zhang H., Liang Z., Yang X., Cao R. (2021). AXL, an Important Host Factor for DENV and ZIKV Replication. Front. Cell Infect. Microbiol..

[17] Munnamalai V., Fekete D.M. (2016). Organotypic Culture of the Mouse Cochlea from Embryonic Day 12 to the Neonate. Methods Mol. Biol..

[18] Munnamalai V., Fekete D.M. (2016). Notch-Wnt-Bmp crosstalk regulates radial patterning in the mouse cochlea in a spatiotemporal manner. Development.

[19] Ruben R.J. (1967). Development of the inner ear of the mouse: A radioautographic study of terminal mitosis. Acta Otolaryngol. Suppl..

[20] Chen P., Segil N. (1999). p27(Kip1) links cell proliferation to morphogenesis in the developing organ of Corti. Development.

[21] Souza B.S., Sampaio G.L., Pereira C.S., Campos G.S., Sardi S.I., Freitas L.A., Figueira C.P., Paredes B.D., Nonaka C.K., Azevedo C.M. (2016). Zika virus infection induces mitosis abnormalities and apoptotic cell death of human neural progenitor cells. Sci. Rep..

[22] Li H., Saucedo-Cuevas L., Regla-Nava J.A., Chai G., Sheets N., Tang W., Terskikh A.V., Shresta S., Gleeson J.G. (2016). Zika Virus Infects Neural Progenitors in the Adult Mouse Brain and Alters Proliferation. Cell Stem Cell.

[23] Sevvana M., Rogers T.F., Miller A.S., Long F., Klose T., Beutler N., Lai Y.C., Parren M., Walker L.M., Buda G. (2020). Structural Basis of Zika Virus Specific Neutralization in Subsequent Flavivirus Infections. Viruses.

[24] Hasan S.S., Miller A., Sapparapu G., Fernandez E., Klose T., Long F., Fokine A., Porta J.C., Jiang W., Diamond M.S. (2017). A human antibody against Zika virus crosslinks the E protein to prevent infection. Nat. Commun..

[25] Miner J.J., Diamond M.S. (2016). Understanding How Zika Virus Enters and Infects Neural Target Cells. Cell Stem Cell.

[26] Nowakowski T.J., Pollen A.A., Di Lullo E., Sandoval-Espinosa C., Bershteyn M., Kriegstein A.R. (2016). Expression Analysis Highlights AXL as a Candidate Zika Virus Entry Receptor in Neural Stem Cells. Cell Stem Cell.

[27] Meertens L., Labeau A., Dejarnac O., Cipriani S., Sinigaglia L., Bonnet-Madin L., Le Charpentier T., Hafirassou M.L., Zamborlini A., Cao-Lormeau V.M. (2017). Axl Mediates ZIKA Virus Entry in Human Glial Cells and Modulates Innate Immune Responses. Cell Rep..

[28] Hamel R., Dejarnac O., Wichit S., Ekchariyawat P., Neyret A., Luplertlop N., Perera-Lecoin M., Surasombatpattana P., Talignani L., Thomas F. (2015). Biology of Zika Virus Infection in Human Skin Cells. J. Virol..

[29] Bhattacharyya S., Zagorska A., Lew E.D., Shrestha B., Rothlin C.V., Naughton J., Diamond M.S., Lemke G., Young J.A. (2013). Enveloped viruses disable innate immune responses in dendritic cells by direct activation of TAM receptors. Cell Host Microbe.

[30] Lemke G., Rothlin C.V. (2008). Immunobiology of the TAM receptors. Nat. Rev. Immunol..

[31] Orvis J., Gottfried B., Kancherla J., Adkins R.S., Song Y., Dror A.A., O’lley D., Rose K., Chrysostomou E., Kelly M.C. (2021). gEAR: Gene Expression Analysis Resource portal for community-driven, multi-omic data exploration. Nat. Methods.

[32] Elkon R., Milon B., Morrison L., Shah M., Vijayakumar S., Racherla M., Leitch C.C., Silipino L., Hadi S., Weiss-Gayet M. (2015). RFX transcription factors are essential for hearing in mice. Nat. Commun..

[33] Liu H., Chen L., Giffen K.P., Stringham S.T., Li Y., Judge P.D., Beisel K.W., He D.Z.Z. (2018). Cell-Specific Transcriptome Analysis Shows That Adult Pillar and Deiters’ Cells Express Genes Encoding Machinery for Specializations of Cochlear Hair Cells. Front. Mol. Neurosci..

[34] Kolla L., Kelly M.C., Mann Z.F., Anaya-Rocha A., Ellis K., Lemons A., Palermo A.T., So K.S., Mays J.C., Orvis J. (2020). Characterization of the development of the mouse cochlear epithelium at the single cell level. Nat. Commun..

[35] Hou S., Chen J., Yang J. (2019). Autophagy precedes apoptosis during degeneration of the Kolliker’s organ in the development of rat cochlea. Eur. J. Histochem..

[36] Kamiya K., Takahashi K., Kitamura K., Momoi T., Yoshikawa Y. (2001). Mitosis and apoptosis in postnatal auditory system of the C3H/He strain. Brain Res..

[37] Locher H., Frijns J.H., van Iperen L., de Groot J.C., Huisman M.A., de Chuva Sousa Lopes S.M. (2013). Neurosensory development and cell fate determination in the human cochlea. Neural. Dev..

[38] Pujol R., Lavigne-Rebillard M. (1985). Early stages of innervation and sensory cell differentiation in the human fetal organ of Corti. Acta Otolaryngol. Suppl..

[39] Leal M.C., Muniz L.F., Ferreira T.S., Santos C.M., Almeida L.C., Van Der Linden V., Ramos R.C., Rodrigues L.C., Neto S.S. (2016). Hearing Loss in Infants with Microcephaly and Evidence of Congenital Zika Virus Infection-Brazil, November 2015–May 2016. MMWR Morb. Mortal. Wkly. Rep..

[40] Thawani A., Sirohi D., Kuhn R.J., Fekete D.M. (2018). Zika Virus Can Strongly Infect and Disrupt Secondary Organizers in the Ventricular Zone of the Embryonic Chicken Brain. Cell Rep..

[41] Meertens L., Carnec X., Lecoin M.P., Ramdasi R., Guivel-Benhassine F., Lew E., Lemke G., Schwartz O., Amara A. (2012). The TIM and TAM families of phosphatidylserine receptors mediate dengue virus entry. Cell Host Microbe.

[42] Wells M.F., Salick M.R., Wiskow O., Ho D.J., Worringer K.A., Ihry R.J., Kommineni S., Bilican B., Klim J.R., Hill E.J. (2016). Genetic Ablation of AXL Does Not Protect Human Neural Progenitor Cells and Cerebral Organoids from Zika Virus Infection. Cell Stem Cell.

[43] Govero J., Esakky P., Scheaffer S.M., Fernandez E., Drury A., Platt D.J., Gorman M.J., Richner J.M., Caine E.A., Salazar V. (2016). Zika virus infection damages the testes in mice. Nature.

[44] Hastings A.K., Yockey L.J., Jagger B.W., Hwang J., Uraki R., Gaitsch H.F., Parnell L.A., Cao B., Mysorekar I.U., Rothlin C.V. (2017). TAM Receptors Are Not Required for Zika Virus Infection in Mice. Cell Rep..

[45] Wang Z.Y., Wang Z., Zhen Z.D., Feng K.H., Guo J., Gao N., Fan D.Y., Han D.S., Wang P.G., An J. (2017). Axl is not an indispensable factor for Zika virus infection in mice. J. Gen. Virol..

[46] Pierson T.C., Diamond M.S. (2020). The continued threat of emerging flaviviruses. Nat. Microbiol.

[47] Renauld J.M., Basch M.L. (2021). Congenital Deafness and Recent Advances Towards Restoring Hearing Loss. Curr. Protoc..

[48] Ficenec S.C., Schieffelin J.S., Emmett S.D. (2019). A Review of Hearing Loss Associated with Zika, Ebola, and Lassa Fever. Am. J. Trop. Med. Hyg..

