# Supplementary material for: Embryonic and Neonatal Mouse Cochleae Are Susceptible to Zika Virus Infection
Source: Viruses. 2021 Sep 14;13(9):1823. doi: 10.3390/v13091823 (PMC8472928; doi:10.3390/v13091823)
Supplement: Supplementary file 1 [file viruses-13-01823-s001.zip › viruses-1355140-supplementary.pdf]

Supplementary Table S1 – Summary of experimental conditions for cochlear cultures

Group	Exp #	Day of explant	Days in vitro	Treatment (number of organs)				Stain			
				Control	ZIKV 24h	ZIKV 48h	AB[1] +ZIKV	dsRNA	Myo6	Sox2	a-cas3
**A - cell types infected**											
	A1	E12.5	6 DIV	4		4		x	x	x	
	A2	E12.5	6 DIV	4		4		x	x	x	
	A3	E12.5	6 DIV	2	3			x	x	x	
	A4	E12.5	6 DIV	3	3			x	x	x	
	A5	E12.5	6 DIV	4	4			x	x	x	
	A6	E15.5	6 DIV	3		3		x	x	x	
	A7	E15.5	6 DIV	2	6			x	x	x	
	A8	E15.5	6 DIV	7	3			x	x	x	
	A9	E15.5	6 DIV	6	3			x	x	x	
	A10	E15.5	6 DIV	5	6			x	x	x	
	A11	P2	6 DIV	0	2			x	x	x	
	A12	P2	6 DIV	2	2			x	x	x	
	A13	P2	6 DIV	4	4			x	x	x	
**B – cell death**											
	B1	E12.5	3 DIV	2	4			x		x	x
	B2	E15.5	6 DIV	2	3			x		x	x
	B3	E15.5	3 DIV	4	1			x		x	x
	B4	E15.5	3 DIV	3	7			x		x	x
	B5	E15.5	3 DIV	4	4			x		x	x
	B6	E15.5	3 DIV	5	3			x		x	x
**C – virus neutralization**											
	C1	E12.5	6 DIV	4	3		4	x	x	x	
	C2	E12.5	6 DIV	3	3		2	x	x	x	

AB[1] = antibody ZIKV-117
